# When I say … workforce sustainability

**DOI:** 10.1111/medu.70072

**Published:** 2025-10-09

**Authors:** Megan E. L. Brown, Eleanor Hoverd, Anthony Montgomery, Bryan Burford, Gill Vance, Workforce Voices

**Affiliations:** ^1^ School of Medicine Newcastle University Newcastle upon Tyne UK; ^2^ Workforce and Learning Research Group, Nuffield Department of Primary Care Health Sciences University of Oxford Oxford UK; ^3^ Department of Psychology Northumbria University Newcastle upon Tyne UK

## Abstract

Workforce sustainability isn't just about filling roles—it's about creating conditions for people to thrive, contribute, and pass on their work. #HPE #Workforce

## 1 INTRODUCTION

An Cailleach, the Gaelic goddess of winter, is said to control the seasons by reshaping the land. In Scotland, for over 5000 years, people have taken part in an unbroken ritual to assist her, by moving stone figures in and out of shelter with the turning year. * This workforce, and its sense of shared purpose, has persisted across nearly 200 generations.

When we say *workforce sustainability,* this is what we mean. Sustainability is not just about retaining bodies in roles but also about sustaining the conditions that enable people to show up, contribute meaningfully and pass on their work and legacy to others. It is about sustaining human effort through time, made possible when cultural and environmental conditions enable meaningful work. Yet, in health professions education (HPE), the phrase ‘workforce sustainability’ is increasingly reduced to discussions of staff vacancy rates, or recruitment targets. We are at risk of workforce sustainability becoming synonymous with presence, rather than seen as an ongoing process that supports human persistence and the environments in which persistence can flourish.

In this ‘When I say’ paper, we reflect on how the term *workforce sustainability* is used within HPE. While increasingly visible in policy and research, the concept is often applied in ways that emphasise metrics before meaning. Drawing on perspectives from HPE, organisational psychology and workforce policy, we argue for a more expansive, context‐sensitive definition that centres the human, relational and institutional conditions that allow a workforce to endure and evolve. Without recalibrating our use of this term, efforts to address workforce challenges risk becoming short‐term and transactional, rather than long‐term commitments to human and systemic renewal.

## 2 WHY WE NEED TO REFRAME WORKFORCE SUSTAINABILITY WITHIN HPE

An effective workforce supports health care delivery, and the learning environments that shape future professionals. In HPE, workforce pressures affect service quality, teaching and educator engagement.^1^ Challenges are not isolated to particular services/settings but reflect broader, systemic strain. As Anderson et al.^2^ argue, persistent vacancies and poor retention reveal the limitations of workforce planning that separates education, training and service.

Montgomery and Lainidi^3^ take this further by highlighting how systems can sustain dysfunction (e.g. cultures of silence) through educational practices. Education is an important way of responding to workforce problems, but it also participates in their construction and reproduction. Habits and expectations formed in training (via formal and hidden curricula) can reinforce existing patterns of organisational behaviour. If education can embed unsustainable ways of working, it can also help nurture alternatives. Understanding workforce sustainability, then, requires attention not only to numbers and roles, but to the values that guide educational and organisational systems, and the mechanisms that maintain or change them.

This is why our collective definition of *workforce sustainability* matters. When used uncritically, the term risks becoming conflated with workforce forecasts/goals, obscuring important dimensions of practice that HPE is uniquely positioned to shape, for example, identity construction, inclusion, relational continuity. Sustainability is not just *of* the workforce, but *within* the workforce. Considering a more holistic definition of workforce sustainability supports us to move beyond short‐term operational fixes via long‐term relational commitment.

## 3 RECONCEPTUALISING WORKFORCE SUSTAINABILITY

In many fields, sustainability is conceptualised using the three‐pillar model originating in the Brundtland Report and the UN Sustainable Development Goals, which emphasises environmental, economic and social dimensions. This model has been applied to HPE and workforce issues by McCormack et al.^4^, who argue that health care workforce sustainability must be understood as part of systemic efforts to safeguard educational continuity and service delivery.

Workforce sustainability is discussed primarily in functional or structural terms, through a ‘survival’ lens focused on vacancy rates, headcounts and short‐term supply. In survival models, sustaining the workforce means simply ensuring enough people are present to meet service needs—staff become manageable resources. This mirrors dominant policy definitions, where human relationships, identity construction and moral responsibility are not central concerns.

In HPE, we believe survival definitions are insufficient, as they ignore the deeper ethical, relational and educational dimensions of clinical work that are shaped, sustained and sometimes distorted through education.^3^ How sustainability feels and unfolds for those within the system remains underexplored. We argue that workforce sustainability should be understood as a quality of systems that support human flourishing over time, rather than as a fixed outcome. These qualities, or conditions, are shaped by policies and infrastructure, but also by the relational and moral fabric of organisations (the values, cultures and interactions that shape whether people feel seen and supported).^3^ They remain underexplored because audit logic and evidence hierarchies privilege what is rapidly countable, dispersing accountability across education–service boundaries and rendering relational labour and moral climate invisible to routine data and policy.^5^

Within our reframing, wellbeing is foundational, not parallel, to sustainability. Panagopoulou and Montgomery^6^ remind us that resilience should be viewed as an organisational, not individual, condition. This shifts attention from personal coping strategies (and perceived ‘deficits’) to the structures, relationships and cultures that keep staff well. Efforts to define a sustainable workforce must centre those most affected: patients and staff. Their experiences are frequently excluded from strategic planning, despite known links between staff wellbeing and patient care.

## 4 CONCLUSION

Within HPE and workforce planning, workforce sustainability is often treated as a neutral metric. We have suggested that sustainability must encompass the conditions that allow people not only to remain in roles but also to flourish within them. Future work should examine these dimensions empirically to support the creation of policy which recognises sustainability as a relational and ethical, as well as structural, concern.

Because language shapes what we measure and value, we offer this working definition as a starting point for deeper reflection and more deliberate use:

*Workforce sustainability is the ongoing capacity to sustain the conditions that enable people to show up, contribute meaningfully, and ensure their work and contributions continue through others, renewing human effort, wellbeing, and purpose across changing systems and time. We understand sustainability not as a fixed outcome, but as a dynamic property of systems, shaped by institutional culture, policy, and societal change*.

The HPE community can serve as workforce stewards by developing this definition, and understanding it empirically with patients, staff and educators. Like myth, sustainability depends on memory and movement. By this, we mean that stones do not move on their own. It is people who return, season after season, to tend what matters.

## Data Availability

Data sharing not applicable to this article as no datasets were generated or analysed during the current study.

## Footnotes

* We draw on the myth of An Cailleach not to universalise or romanticise cultural heritage but to reflect on a story from the land of some of the authors of this paper. We are mindful that myth, landscape and tradition are not neutral, and we aim to use this reference respectfully, recognising both the long history of collective human effort it represents, and the local meanings it carries.
